# Insertion sequence content reflects genome plasticity in strains of the root nodule actinobacterium *Frankia*

**DOI:** 10.1186/1471-2164-10-468

**Published:** 2009-10-12

**Authors:** Derek M Bickhart, Johann P Gogarten, Pascal Lapierre, Louis S Tisa, Philippe Normand, David R Benson

**Affiliations:** 1 Department of Molecular and Cell Biology, U-3125, University of Connecticut, Storrs, CT, USA; 2 Department of Microbiology, University of New Hampshire, Durham, New Hampshire, USA; 3 Université de Lyon, Unité Mixte de Recherche, Centre National de la Recherche Scientifique (UMR CNRS), 5557 Ecologie Microbienne, IFR41 Bio Environnement et Santé, Université Lyon I, Villeurbanne 69622 cedex, France

## Abstract

**Background:**

Genome analysis of three *Frankia sp. *strains has revealed a high number of transposable elements in two of the strains. Twelve out of the 20 major families of bacterial Insertion Sequence (IS) elements are represented in the 148 annotated transposases of *Frankia *strain HFPCcI3 (CcI3) comprising 3% of its total coding sequences (CDS). EAN1pec (EAN) has 183 transposase ORFs from 13 IS families comprising 2.2% of its CDS. Strain ACN14a (ACN) differs significantly from the other strains with only 33 transposase ORFs (0.5% of the total CDS) from 9 IS families.

**Results:**

Insertion sequences in the *Frankia *genomes were analyzed using BLAST searches, PHYML phylogenies and the IRF (Inverted Repeat Finder) algorithms. To identify putative or decaying IS elements, a PSI-TBLASTN search was performed on all three genomes, identifying 36%, 39% and 12% additional putative transposase ORFs than originally annotated in strains CcI3, EAN and ACN, respectively. The distribution of transposase ORFs in each strain was then analysed using a sliding window, revealing significant clustering of elements in regions of the EAN and CcI3 genomes. Lastly the three genomes were aligned with the MAUVE multiple genome alignment tool, revealing several Large Chromosome Rearrangement (LCR) events; many of which correlate to transposase clusters.

**Conclusion:**

Analysis of transposase ORFs in *Frankia *sp. revealed low inter-strain diversity of transposases, suggesting that the majority of transposase proliferation occurred without recent horizontal transfer of novel mobile elements from outside the genus. Exceptions to this include representatives from the IS3 family in strain EAN and seven IS4 transposases in all three strains that have a lower G+C content, suggesting recent horizontal transfer. The clustering of transposase ORFs near LCRs revealed a tendency for IS elements to be associated with regions of chromosome instability in the three strains. The results of this study suggest that IS elements may help drive chromosome differences in different *Frankia *sp. strains as they have adapted to a variety of hosts and environments.

## Background

The genus *Frankia *consists of actinobacteria that form root nodule symbioses with non-leguminous plants wherein they fix N_2 _to ammonia that is assimilated by the plant [1-4]. The genomes of three *Frankia *sp. strains show a complex pattern of deleted, duplicated and hypothetical genes plus many transposable elements suggesting a high degree of plasticity[5]. Despite having 16S rRNA sequences that are greater than 97% identical to each other, the strains have genome sizes that range from five to nine Mbp in size. Their genome sizes reflect the diversity of plants infected [5]. HFPCcI3 (CcI3), with the smallest genome (5.4 Mbp) of the three, infects plants from one family whereas EAN1pec (EAN), with the largest genome (9 Mbp), infects plants in five families. Strain ACN14a (ACN) has a moderate genome size (7.4 Mbp) and infects plants from two families [1].

The number of transposase open reading frames (ORFs) in the *Frankia *genomes is not proportional to their sizes, contrary to some models suggesting that larger genomes are likely to contain more mobile genetic elements than smaller genomes [6]. Initial annotations have indicated that transposase ORFs, associated with insertion sequences (ISs), are highly duplicated and diverse in strains CcI3 (148 orfs) and EAN1 (183 orfs), but less so in strain ACN14a (33 orfs).

IS elements are mobile genetic elements that lack a selectable marker gene and insert into the genome of a host without the need for extensive DNA homology at a target site (for a review see [7-10]). Most bacterial IS elements consist of one, or more, transposase ORF(s) that catalyze excision from, and reinsertion into, a genome. They are classified into major families based on amino acid sequence similarity, structure of ORFs devoted to transposition or the presence of flanking repeat sequences [11]. Inverted repeats (IRs) are commonly found flanking the transposase ORF(s) of IS elements, but are notably absent in the IS200/IS605 [12-14] and IS110 families [15]. IRs can contain promoter elements for the flanked ORF(s) and serve as targets for the active transposase [11,16,17]. Transposition often creates small direct repeats (DR) beside the IR sequences of the element, which may be used to identify prior insertion points of that element in the genome.

IS-associated transposase genes are generally poorly expressed [18]. The insertion, excision, or duplication of IS elements can cause insertion mutations or lead to genome rearrangements often, but not always, to the detriment of the host [7,19]. Point mutations that make IS elements hyperactive can be lethal to their host [20-22]. If many ISs are maintained within a genome, they may confer a degree of genome plasticity allowing for rapid adaptation to new environments [6,23,24].

Insertion sequences have also been implicated as causes of large chromosome rearrangements through intra-chromosomal recombination [7,25,26]. Such changes might allow some pathogens to evade or adapt to host defences [27,28]. Large numbers of IS elements have been shown to induce genome deletions and rearrangements in the pathogens *B. pertussis *and *B. parapertussis *[29]. Similarly, a hostile or changing soil environment could select for microbes that can quickly adapt to new conditions. It is also possible that IS elements confer no such selective advantage on their hosts. This study focused on annotated transposase ORFs in *Frankia *sp. and the identification of fragmentary IS elements present in three strains of *Frankia *sp. Their positioning and diversity suggest roles in driving genome size and strain differences that may have contributed to the adaptation of different *Frankia *lineages to their hosts and soils.

## Results

### Classification of IS content

A total of 364 IS-associated transposase ORFs have been annotated among the three *Frankia *genomes. *Frankia alni *strain ACN has 33, *Frankia *sp. strain CcI3 has 148 and *Frankia *sp. strain EAN has 183 ORFs distributed among 13 IS families plus several unclassified transposases (Table 1 and [5]). This diversity of transposase ORFs is the greatest yet found in bacterial genomes as of this time. The 33 ISs in ACN are in nine families plus the unclassified ORFs. The 148 ORFs in CcI3 are in 12 of the 13 *Frankia *IS families. Four paralog groups of transposases in CcI3 contain ORFs that, within each group, have >99% amino acid sequence identity; the largest group contains 14 IS4 transposase ORFs that share 100% amino acid identity. These paralog groups reflect probable recent duplication [30]. The 183 ORFs in EAN are even more diverse with members in all families with few that are identical. Inverted repeats were found flanking 16 transposases in EAN and 41 transposases in CCI3 (see Additional File 1: Papersupplemental1.xls).

**Table 1:** IS family diversity in three *Frankia *strains

**IS Family^1^**	**Unique to CcI3**	**Unique to EAN**	**Unique to ACN**	**In EAN and CcI3 only^2^**	**In ACN and either EAN or CcI3^3^**	**In all three**
IS3	2	11	-	-	-	-

IS4	7	10	-	63	1	25

IS5	-	-	-	2	-	-

IS6	-	1	-	1	-	1

IS30	-	-	-	2	-	1

IS66	-	-	-	16	-	1

IS110	-	9	-	5	1	7

IS200	-	2	-	8	-	-

IS605	-	9	-	6	-	13

IS630	-	-	-	4	1	15

ISL3	1	-	-	-	-	5

Mutator	-	5	-	-	-	-

Tn3	-	-	-	1	2	3

Unclassified Transposase	4	16	5	35	7	32

Cutoff^4^	15	6	3	-	-	-

Total	29	69	8	143	12	103

^1 ^Determined from annotationing transposase ORFs for EAN and CcI3. ACN transposases were reannotated after the BLAST search to show IS family diversity of transposase ORFs, as all transposases were originally annotated as "putative." Transposases in EAN and CcI3 were not reannotated.

^2 ^Transposase ORFs that hit other ORFs in EAN and CcI3 but not in ACN.

^3 ^Transposase ORFs that hit other ORFs in ACN and one of the other two strains, but not in all three *Frankia *strains.

^4 ^The number of transposase ORFs in each strain that did not hit any sequence in the nr database with an E-value smaller than 10^-15^. In all cases this was due to a sequence size of less than 80 amino acids.

**Figure 1:**
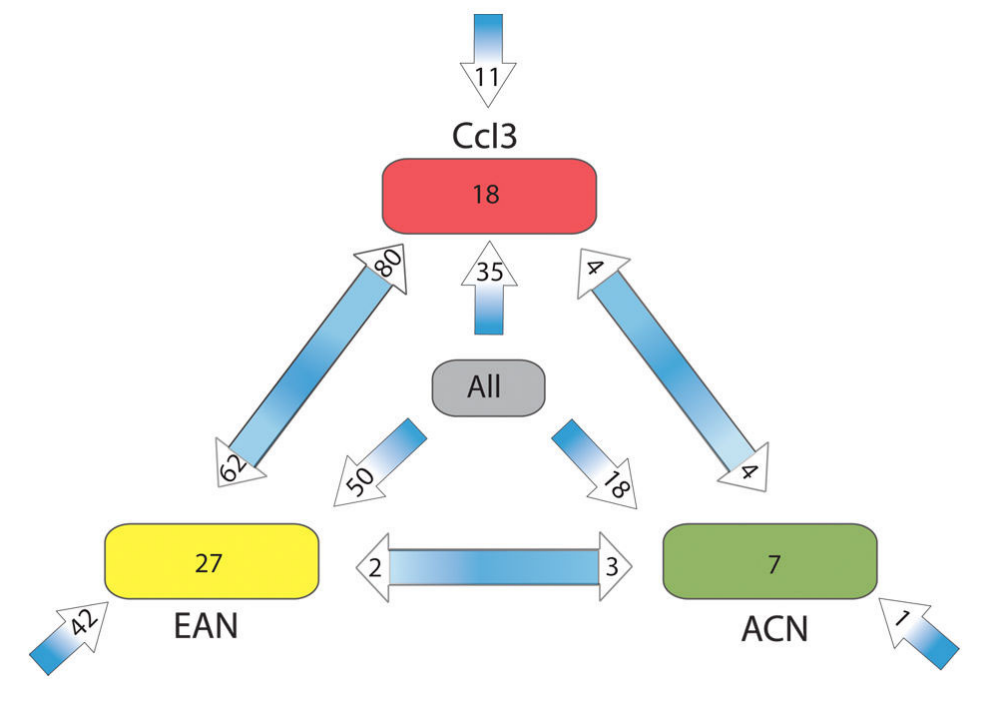
**Transposase ORF distribution in *Frankia *sp**. Putatively shared ORFs were found using BLAST searches of each transposase ORF against the non-redundant (nr) database. Numbers on the arrow heads closest to the strain buttons indicate the number of transposase ORFs that it had in each homologue category. Double headed arrows represent ORFs that had BLAST hits between two strains of *Frankia *sp. The three innermost arrows pointing away from the button labelled "all" indicate the number of transposase ORFs from each strain that hit ORFs in both of the other strains. The three outermost arrows represent the number of ORFs in each strain that only had BLAST hits in other species. The numbers inside the buttons for each strain indicate the transposase ORFs that had no BLAST hits above an E-value of 10^-15^.

For identifying transposases from ancient or horizontal transfer [31] we conducted BLAST searches of the non-redundant (nr) database using the amino acid sequences of all annotated *Frankia *transposases. Most of the ORFs (258, 70.8%) hit transposase ORFs in at least one other *Frankia *strain (Table 1). Of these, 103 ORFs had BLAST hits among all three *Frankia *strains, with 18 (55%) of such ORFs in strain ACN, 35 (23%) in CcI3 and 50 (27%) in EAN. Only 8, 29 and 69 ORFs limited to ACN, CcI3, or EAN respectively (Figure 1). Fifty-five of those ORFs hit transposases (Evalue < 10^-15^) in other bacteria but not in the other strains of *Frankia*; 42 of those ORFs belong to strain EAN. A complete listing of the results of the BLAST searches against the nr database can be found in Additional File 1: papersupplemental1.xls.

**Figure 2:**
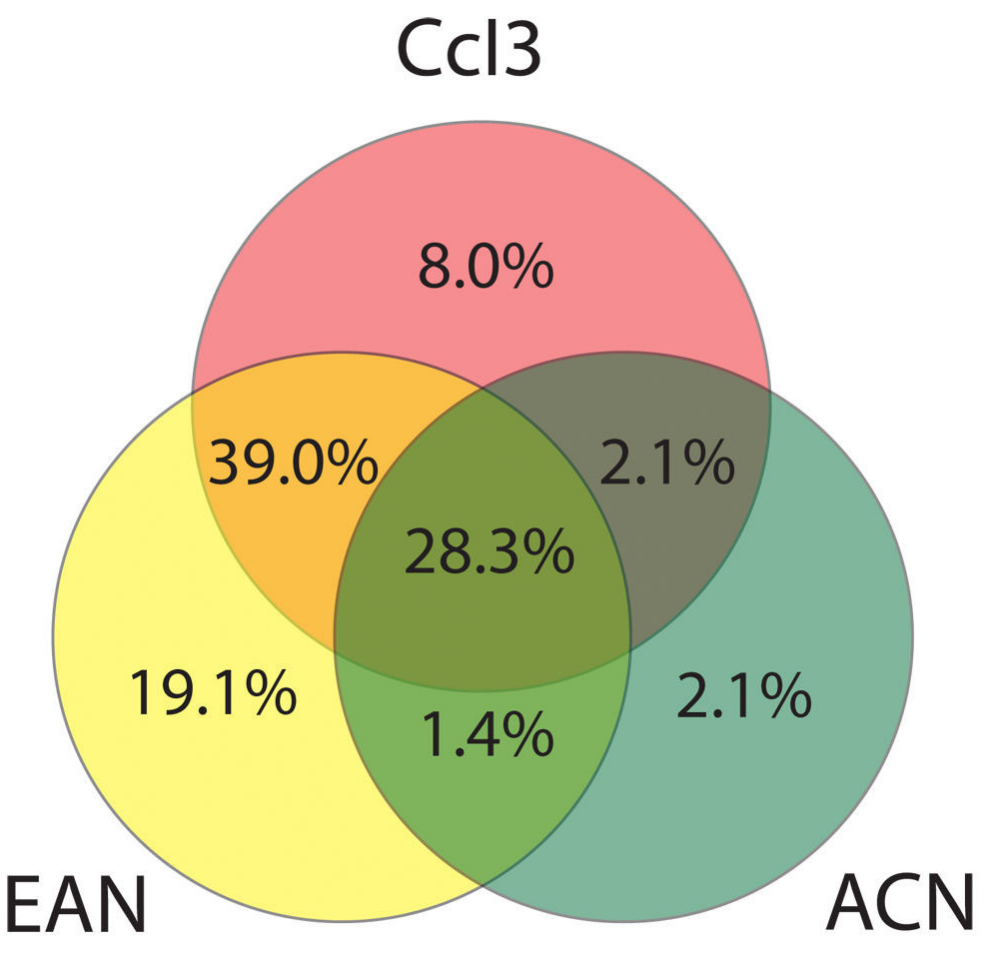
**Percent of shared IS content in *Frankia *sp**. Shared transposase ORFs as a percent of the total number of transposases annotated in *Frankia *sp (364 ORFs). The majority of ORFs (~67.3%) are shared by strains CcI3 and EAN. Only 29.2% of all transposase ORFs are found in only one strain, with 19.1% unique to EAN alone. This distribution suggests that the majority of transposase ORFs have been maintained by and have proliferated within the *Frankia *strains despite geographic isolation.

Interestingly, the largest shared group was composed of 80 transposase sequences in CcI3 that retrieved sequences in EAN (Figure 1). The second largest group was comprised of 62 ORFs in EAN that hit ORFs in CcI3, making 142 total ORFs shared by CcI3 and EAN. Altogether, more than 67% of all *Frankia *sp. transposases are present in groups shared between strains CcI3 and EAN (Figure 2). The ACN genome contains only 9% of the total number of *Frankia *sp. transposases but shares most (18/33; 55%) of those ORFs with the other two strains. Eight (24.4%) transposase ORFs in ACN are unique to the strain, compared with 38% (69) of the transposase content of EAN. ACN also lacks the diversity of IS content of the other strains with members of only nine IS families. The majority of novel transposase ORFs found in strain ACN are putative; suggesting that they may be remnant members of other IS families. Ten of the eleven members of the IS3 family in strain EAN have no external BLAST hits at the 10^-15 ^cut-off value.

In order to screen for transposases that may have been more recently horizontally transferred, we compared transposase ORF G+C% to the results of the BLAST searches. The three *Frankia *genomes have a combined average G+C% of 71.28% [5], so we considered a G+C% of less than 65% to be significantly below the average. Using this cut-off value, we identified 5, 6 and 23 transposase ORFs that had significantly lower G+C content in strains CcI3, ACN and EAN respectively. Members of the IS3 family that are novel to strain EAN make up 39% (9) of the 23 low G+C% transposase ORFs identified in its genome. This fact supports the notion that a majority of the IS3 family transposases were a horizontal acquisition by strain EAN. Excluding the IS3 family in strain EAN, relatively few strain specific transposase ORFs had low G+C percentages. Only three other transposases (one per strain) had a low G+C percentage in addition to no external BLAST hits. Seven of the 23 low G+C transposases were identified as being shared by all three strains, with 1, 2, and 4 ORFs present in strains CcI3, ACN and EAN respectively. All seven ORFs were annotated as IS4 transposases with an average length of 390 amino acids.

### PSI-TBLASTN Analysis of Genomes

Position specific scoring matrices (PSSM's) were generated for the five major IS families found in the original annotation of the three *Frankia *genomes. Amino acid sequences of all IS110, IS4, IS605, IS630 and IS66 transposases found in the *Frankia *genomes were used to create five separate PSSM's for each major family. To reduce potential false positives, only two iterations of the PSI-BLAST algorithm were run against the non-redundant database for each PSSM. Each PSSM was then used in separate TBLASTN searches against the nucleotide sequence of each genome. The results of these searches uncovered 36% (53) and 42% (77) more transposases or their remnants in strains CcI3 and EAN, respectively (Table 2). Only four putative transposase remnants were identified in strain ACN.

**Table 2:** Results of PSI-TBLASTN for top 5 transposase families

**Category**	**ACN**	**CcI3**	**EAN**
Initial number ^1^	13	102	98

Hits Identified	17	160	170

True Positives	17	154	165

Intergenic remnants (by PSI-BLAST) ^2^	2	21	32

Reannotated ^3^	2	32	45

False Positives ^4^	0	6	5

False Negatives	0	0	0

^1 ^Number of original annotated transposase ORFs from the five major IS families (IS4, IS110, IS66, IS630 and IS605) that were used in the PSI-BLAST.

^2 ^Hits that involved a majority of nucleotides that were in between annotated ORFs.

^3 ^ORFs that were not initially annotated as transposases of the PSSM IS family but were hits of the PSI-BLAST search. This included ORFs that were annotated as putative transposases.

^4 ^Hits that were lower than 40% ID and/or less than 50 bp in length.

Several transposase remnants were identified in intergenic regions, including 21 fragments in CcI3 and 32 in EAN. The remaining putative transposases were previously annotated ORFs that were reclassified as transposases by the PSI-TBLASTN search, with 45 in EAN and 33 in CcI3. One ORF reclassification of note is the reassignment of a family of cytosine-5-methyltransferases as transposases of the IS605 family in EAN (see Additional File 2: PsiBlastSupplemental.xls). Conserved domain feature identification using pre-recorded data on the NCBI site reveals that this family of methyltransferases contains two transposase-associated protein domains and no methyltransferase domains. The presence of transposase protein domains in these ORFs supports their reclassification as IS605 transposases.

Due to the stringency of the search, each novel putative transposase was identified by only one PSSM; however, some hits from the search gave different nucleotide coordinates for the same putative transposase ORF that varied by as much as 100 bp. The hit with the highest percent identity to the query sequence was retained in those situations. False positives were identified as sequences having less than 50 bp of length plus less than 40% amino acid identity to the query sequence. Five false positives were identified in strain EAN and six in strain CcI3. All originally annotated transposases of the five major IS families used in the PSI-TBLASTN search were identified, resulting in no false negatives. Altogether, a total of 37 transposase ORFs plus remnants were identified in ACN, 201 were identified in CcI3 and 261 in EAN. The complete list of reannotated ORFs and truncated derivatives can be found in Additional File 2: PsiBlastSupplemental.xls.

### Identification of Large Chromosome Rearrangements

Using the MAUVE genome alignment program [32] we identified 8 and 13 Large Chromosome Rearrangements (LCRs) in strains CcI3 and EAN respectively using ACN as the reference (Additional File 3: PaperSupplemental3.xls). We defined the LCRs as regions greater than 5 kb common to all three strains with a maximum of a 100 bp gap that were out of order or inverted compared to homologous regions in strain ACN. A preliminary GRIMM (Genome Rearrangement In Man and Mouse) analysis [33] of our original MAUVE alignment projected that ACN had the fewest rearrangement events from the node of the trifurcation (data not shown).

**Figure 3:**
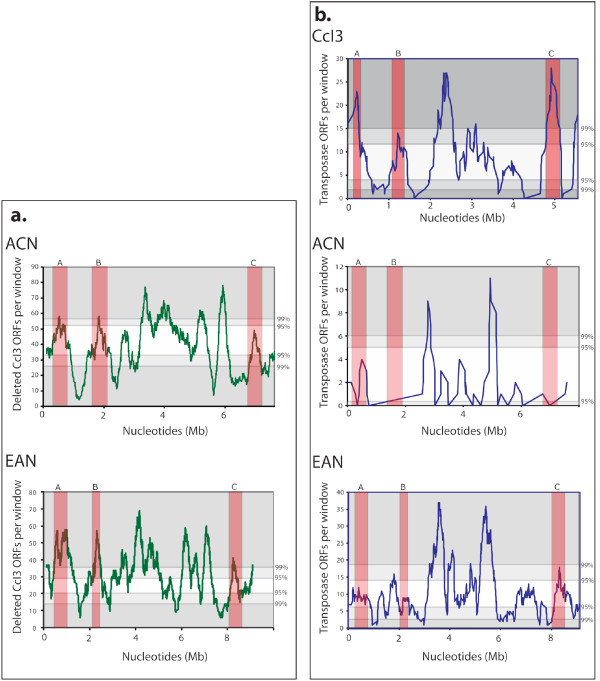
**CcI3 gene deletions and IS clustering**. (a) Genes that were deleted in strain CcI3 but were present in both EAN and ACN were plotted using a 250 kb sliding window (dark green line). (b) Transposase ORF positions (including those identified by PSI-BLAST analysis) were plotted using a 250 kb sliding window for each strain (dark blue line). Regions of each genome that corresponded to significant clusters of gene deletion in strain CcI3 are highlighted and lettered (red boxes). Confidence intervals determined from calculation of the probability mass function are listed on the right of the graphs, with points greater than 95% confidence in light gray boxes and points greater than 99% confidence in dark gray boxes.

Interestingly, nearly all LCRs in strains CcI3 and EAN occur either near the origin of replication of the chromosome or at its terminus. Inversions at the terminus can be explained by recombinase-mediated homologous recombination [34], however, overlays of transposable element density maps on the genomes revealed IS clustering near these regions as well, potentially linking terminus inversions to intra-chromosomal recombination of mobile genetic elements (Figure 3). Despite evidence of clustering in these regions, identical transposase ORFs do not flank any identified LCRs as was found with a large genome inversion mediated by IS905 in *Lactococcus lactis *[35]. Chi square analysis of transposase gene content within each defined LCR segment revealed no significant difference from expected numbers of ORFs for a region of the same size (Additional File 3: PaperSupplemental3.xls). This suggests that the LCRs themselves do not serve as hotspots for IS element insertion, despite their rearrangement with respect to strain ACN. Instead, they appear to be relatively stable genomic islands that have simply moved to new loci within each genome.

### Insertion Sequence Clustering

Previous heuristic analyses of the three *Frankia *genomes found more transposase ORFs in regions of each genome lacking synteny with the others [5]. In order to analyze these IS hotspots, a sliding window was used to count the number of transposase ORFs present every 250 kb. All transposase positions found in our PSI-TBLASTN search were used in the data sets for each organism, as the data recovered from that analysis most likely indicates recent as well as current positions of IS elements in the genomes. To assess statistically the distribution of IS elements, we used a probability mass function (pmf) derived from the average number of transposases found per window for each genome. That number varied from 3, to 12 to 10 ORFs in ACN, CcI3, and EAN respectively.

Strain ACN was found to have significant clustering of transposase ORFs near 3 and 5 Mb (Figure 3b). Despite the relatively smaller dataset compared to the other two strains, these clusters in ACN represented 9 and 11 transposases in a 250 kb window giving their clustering a greater than 99% confidence. The ACN genome also has significant clustering of transposase ORFs near the terminus mirroring the heterogeneity of the terminus found in all three genomes.

**Figure 4:**
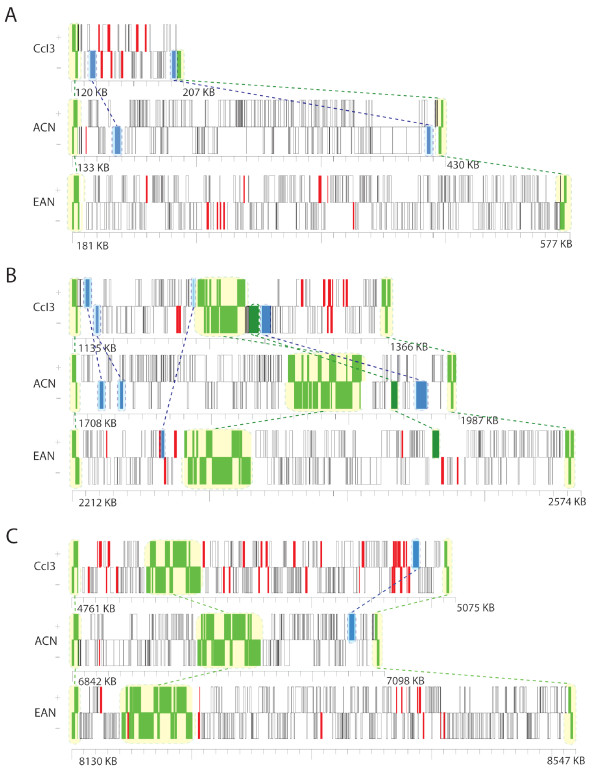
**Neighbourhood analysis of highlighted windows in Figure 3**. Each ORF is depicted as a box. Boxes on top are transcribed from the forward strand (+). Those on the bottom are transcribed from the reverse strand (-). ORFs common to all three strains are highlighted in green. Transposases are in red, and ORFs that are only common to two strains are in blue. ORFs that were not present in the other genomes in each window are in white boxes. These ORFs were either present in the other genomes in different loci, or were unique to strains ACN and EAN and were not present in strain CcI3. Dashed lines indicate points of reference between the three genomes.

Approximately 57% of all transposase ORFs identified in strain CcI3 are found either near the origin, or between the 2 Mb to 2.5 Mb region. Both of these regions have clusters well above a 95% confidence interval, suggesting that the clusters are not solely due to random insertion. These regions are in proximity to LCRs in the CcI3 genome, suggesting preferential IS insertion around these regions may be linked with *Frankia *genome rearrangement events. Again, the terminus of this genome serves as a hotspot of transposase ORFs in addition to a large peak near the 4.9 Mb region of the genome closer to the origin. This region of the genome was found to correspond to a potential deletion (Figures 3 and 4).

The number of transposase ORFs found in the EAN genome dwarfs those found in the other two strains; a clear pattern of clustering was found. Two major clusters were near and symmetrically oriented around the terminus near 3.5 and 5.5 Mb. Both clusters had a confidence interval greater than 99%, and corresponded to breaks in synteny with the ACN genome. The lack of synteny near these clusters suggests that these regions may contain gene duplicates. An increased presence of the top three duplicated gene families in strain EAN near these clusters confirms this prediction (Table 3). The symmetrical appearance of these clusters was striking but their significance remains unknown.

**Table 3:** Number of Transposases in Breaks in Synteny

**Strain**	**Area^1^**	**Transposases^2^**	**p value^3^**	**Duplicated genes^4^**
EAN	5964668 (66%)	223 (86%)	1.28 × 10^-3^	262 (306) *

CcI3	2418797 (45%)	134 (67%)	2.18 × 10^-6^	18 (28)

^1 ^The number of nucleotides in regions of the strain that did not show MAUVE alignment synteny with strain ACN in a continuous 10 kb+ stretch. Numbers in parentheses indicate the percentage of the genome to which these stretches correspond.

^2 ^The number of transposase ORFs in the breaks in synteny. Numbers in parentheses indicate the percentage of all transposases in the strain that were found, including those identified in the PSI-TBLASTN search.

^3 ^P value derived from a Chi squared test of the number of transposases in the region against an expected number.

^4 ^The number of the top 3 duplicated gene family ORFs in each respective strain that were not transposases in this region. Numbers in parentheses are the total number of duplicated genes of the three top duplicated gene families in that strain. (*) indicates a p value less than 0.005.

### Gene Deletion in strain CcI3

Using a similar sliding window analysis, we mapped ORFs present in strains EAN and ACN but deleted in strain CcI3 [5]. A comparison of this map (Figure 3a) with the transposase cluster map (Figure 3b) revealed a pattern of gene deletion corresponding to some clusters of transposase ORFs in the CcI3 genome. Comparing all ORFs in these regions revealed a general lack of synteny in all three strains, with a notable reduction of both nucleotides and number of ORFs in strain CcI3 in comparison to EAN. Three such regions flanked by syntenic LCBs were selected for a more detailed comparison of gene position and content among the three strains (Figure 4). Regions A and B have fewer ORFs in CcI3's genome compared to ACN; region C showed an increase in sequence over ACN. Only two transposase ORFs were present in strain ACN over all three regions. CcI3 had 8, 10 and 35, and EAN had 10, 7 and 14 transposase ORFs in regions A, B and C, respectively.

The termini of ACN and EAN have the largest clusters of genes deleted in CcI3; however, a general lack of synteny among all three strains in this region precluded a neighborhood analysis. In general, the largest significant cluster of transposases from the 2 Mb to 2.5 Mb region of strain CcI3 corresponds to regions of gene deletion.

## Discussion

### *Frankia *IS content

Despite having transposase ORF's from thirteen major IS families, relatively few are strain-specific in *Frankia*. The presence of diverse and numerous IS elements in a genome is often believed to reflect horizontal gene transfer [6]. Given the different geographic origins of the three strains [1,5] and the sequence similarity of their transposase ORFs, it is unlikely that recent horizontal gene transfer is solely responsible for the proliferation of transposases in strains EAN and CcI3. Instead, it appears that the majority of transposase ORFs are descended from ones that were present in a common ancestor that diverged with the emergence of actinorhizal plant families approximately 100 million years ago [36]. Some proliferated or were maintained in lineages leading to strains EAN and CcI3, but some were lost from the ACN lineage. One family of transposases, the IS3 transposases of EAN, and a small subset of IS4 transposases are clearly horizontally acquired. These 16 ORFs only comprise ~5% of the total number of transposases annotated. The general lack of novel IS elements is further supported by the near absence of unique transposase ORFs in ACN.

Remnant transposases, as detected through the PSI-TBLASTN method, indicate past IS activity. The low ratio of annotated IS elements to newly discovered fragments in strain ACN suggests that IS movement was selected against in this lineage. By contrast, the EAN genome has at least 81 transposase remnants, suggesting that transposition and/or duplication are common and ongoing. Transposase ORFs are associated with heterogeneity and expansion at the terminus of the EAN genome, as compared to the ACN genome, as well as the large number of LCRs identified by the MAUVE alignment. IS elements have long been known to facilitate chromosome rearrangements [7,28,37,38] so breaks in synteny near identifiable IS clusters are likely due to IS-mediated rearrangements. Support for such activity outside of observed proximity would require direct observation of chromosome rearrangement.

### Strain ACN

With only 33 identifiable transposase ORFs in a genome of 7 Mb, the ACN genome has fewer transposases than the other two *Frankia *genomes. Given the potentially deleterious nature of IS elements [6,7], one would expect that transposition events would be fixed infrequently in a population. In phylogenetic analyses, CcI3 and ACN are more closely related than either is to EAN [5]. Thus, the presence of nearly identical transposase ORFs in strains EAN and CcI3, but not in ACN suggests that the common ancestor of all three strains contained elements from many IS families that were retained and duplicated in lineages leading to EAN and CcI3 but lost in ACN. All lineages continued to acquire novel ISs through horizontal transfer.

### Genome expansion of strain EAN

In contrast to strain ACN, 77 remnant and 182 annotated transposases are found in the EAN genome. The transposase ORFs identified in this study amount to approximately 3% of the EAN genome, and nearly 4% of the total CDS. The high numbers of IS elements may help strains in the EAN lineage adapt quickly to new niches. A similar observation has been made in clinical isolates of *Enterococcus faecium *in which IS elements were implicated in developing new subspecies that are better adapted to a hospital environment [23]. Of the three sequenced strains, members of the EAN lineage infect the broadest range of host plant families [1]. This broad host range may be enabled by gene duplications, and rearrangements, driven by IS element insertion and cointegrate formation. At present, 123 pseudogenes have been identified in the finished assembly of the EAN genome. Similar pseudogene and IS element content is found in *Yersinia pestis*, another dynamic genome [39].

**Figure 5:**
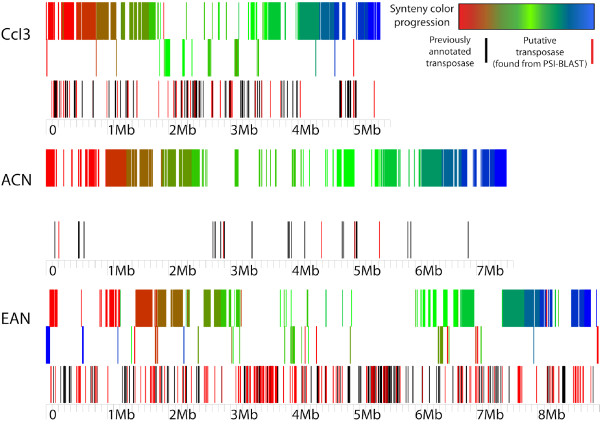
***Frankia *sp. genome alignment with transposase clustering**. MAUVE backbone files were imaged using the Genvision™ Adobe Illustrator^© ^plug-in to show distinct LCR events. The top of each genome's histogram represents syntenic LCB's that are in the same order with respect to the ACN genome. The middle layer represents LCB's that are inverted with respect to ACN. The bottom histogram shows positioning of originally annotated (black) and PSI-BLAST determined (red) transposase ORFs in each genome.

IS clusters determined by our sliding window analysis may indicate genome instability in certain regions of the EAN genome. At least two statistically significant IS clusters were identified; one is almost entirely composed of transposases identified by the PSI-TBLASTN search (red bars in Figure 5). We also identified regions of the EAN genome that have expanded by gene duplication (Figure 4). An increased number of ORFs and other nucleotide sequence in regions that are comparatively deleted in strain CcI3 suggest that such regions in the *Frankia *genomes have been subject to major genome polymorphisms.

### Genome reduction of strain CcI3

The genome of CcI3 appears to be undergoing genome ratcheting coincident with specialization within a narrow range of plant hosts and soil types [5]. IS clusters, consisting largely of those present in the common ancestor of the three *Frankia *strains, may have promoted the deletion of large regions around the terminus during homologous recombination (Figure 3). This is evidenced by the paucity of non-transposase gene duplicates in strain CcI3 compared to strain EAN and ACN [5], as well as by the presence of 53 fragmentary transposases identified by the PSI-TBLASTN analysis. Analysis of regions of the CcI3 genome that have lost ORFs with respect to the other two strains has shown a loss of DNA in two regions of the genome identified from the sliding window plot (Figure 4). The presence of clusters of transposase ORFs in these regions suggest that the IS elements were either responsible for the deletion events, or capitalized on the fragility of this hotspot. Analysis of the *Frankia *proteome using LC MS/MS has recovered peptide fragments associated with five transposases present in deletion windows A (3) and B (2). Peptides of nine other transposase present at the terminus of the CcI3 genome were also identified (J.E. Mastronunzio and Y. Huang. personal communication). Given that transposases can have numerous barriers to expression during transcription and translation [17,40,41], finding transposase peptides suggests that transposases remain active in the *Frankia *genomes.

Most transposase ORFs in CcI3 have homologs in the other strains, so the emergence of a strain of *Frankia *that infects plants from a single family appears to have been accomplished primarily through the activity of native mobile genetic elements. Whereas EAN enjoys a large host plant family range and a geographically diverse distribution, CcI3's original native range incorporates parts of Australia and Pacific islands [42]. Without diverse selective pressures, intramolecular recombination may have led to the reduction of the CcI3 genome and a narrower host range [43].

## Conclusion

In the three sequenced *Frankia *genomes, IS proliferation has occurred largely without the recent input of IS elements from horizontal gene transfer. Clustering of existing transposases in the genomes is statistically significant and corresponds to regions lacking synteny with the other two strains. This finding suggests that such sequences within strains CcI3 and EAN have contributed to genome contraction and gene duplication events respectively. While it may seem contradictory to propose that IS elements have catalyzed both expansion and reduction within the *Frankia *genomes, selective pressures in conjunction with environmental niche availability appear to be nudging the genomes in opposite directions.

## Methods

### Sequence Manipulation

The annotations and amino acid sequences for the transposase genes for *Frankia *strains CcI3 [genbank:CP000249], EAN [genbank:CP000820] and ACN [genbank:CT573213] were obtained from the NCBI Genbank website . A useful resource for IS classification and nomenclature is the ISfinder website  but *Frankia *ISs are only partially classified in the most recent version. FASTA files of *Frankia *transposase amino acid sequences obtained from Genbank were used in a BLAST [44] search of the non-redundant (nr) protein database. The E-value cutoff was set at 10^-15^in order to account for smaller transposase sequences. Reciprocal hits of the smaller transposase amino acid sequences often generated E-values of less than 10^-20^.

Only the amino acid sequence of each transposase ORF was used due to the actinobacterial tendency to replace the third base pair of each codon with a guanine or cytosine residue [1,4]. This tendency also prevented a dn/ds analysis of the transposase orfs as is traditionally used in studies of this type to determine purifying selection against transposable elements [45]. Only annotated transposase orfs were used in this analysis. Inverted repeats were also omitted from the analysis as they do not contribute to the amino acid sequence of the transposase protein.

### Inverted Repeat Identification

IS element inverted repeats were identified using the Inverted Repeat Finder (IRF) program version 2.17 [46]. Initial IRF processing was performed on the genomes of the three *Frankia *strains CcI3, ACN14a and EAN1 using the parameters 2, 3, 5, 40 (match, mismatch, indel, minimum score). The k-tuple values used in this initial processing corresponded to 500 for the T4 tuple class, 2000 for the T5 tuple class and 10000 for the T7 tuple class. The "lookahead test" and the "third alignment going inwards" options were also selected for the genome processing in order to more stringently identify IR sequences.

### Identification of Putative or Fragmentary IS Elements

Fragmented IS elements were identified using a PSI-TBLASTN approach [47,48]. Position specific scoring matrices (PSSM's) were created using the amino acid sequences of annotated *Frankia *sp. transposases as queries in BLAST searches against the nr database in two PSI-BLAST iterations. Separate PSSM's were made for each annotated IS family. Searches were then performed on a six-frame translation of the nucleotide sequence of each respective genome using the TBLASTN program. Results were sorted based on sequence percent identity to the query. Hits with percent identities lower than 30% were discarded, as were hits with less than 50 bp in sequence length.

### Multiple Genomic Alignments

Genomes were aligned using the MAUVE genome alignment program version 2.1.1 [32]. Original alignment of the genomes was done with a minimum backbone setting of 100 bp. Local colinear block (LCB) weight of this alignment was increased until only 29 LCB's were identified by the program. LCB order numbers were then input to the GRIMM web server for rearrangement modeling [33] to determine which genome had fewer rearrangement events from the node of the trifurcation. Strain ACN was determined to have fewer LCR events and was used as an alignment standard. Subsequent MAUVE alignment of the genomes used a minimum backbone of 1200 bp, with a 500 bp minimum island value to eliminate spurious LCB assignments. Backbone and island files were visualized using the Genvision plugin for Adobe Illustrator^©^.

### Statistical Correlation for IS and Gene Deletion Clustering

A 250 kb sliding window was used to determine the total number of elements present within each window of the genomes (accounting for their circular chromosomes). The nucleotide mid-point of each element was used as the position of the element. For ease of manipulation, only window positions where there were changes in the number total of features were used to generate the distribution figures. Using the average number of elements per window, the probability of finding particular number of elements under a Poisson distribution was determined by using the probability mass function (pmf).



Where "*λ*" is the average number of elements per window and "*k*" is the number of elements in a given interval. Genes that were predicted to have been deleted in strain CcI3 yet were still present in both ACN and EAN were mapped using another 250 kb sliding window. Windows in which the number of CcI3 deleted genes rose above the upper 99^th ^percentile in either ACN or EAN were compared against MAUVE alignments of all three genomes. If any region had a significant cluster of deleted genes and was found between syntenic LCB's, it was depicted in Figure 4. Three such regions were identified, despite other regions of significant clustering of deleted genes.

## Authors' contributions

PL performed the sliding window analysis of deleted genes and transposase ORFs and provided technical support in the use of several bioinformatics programs. JPG, LST, PN and DRB provided the intellectual framework of the study and recommended several bioinformatics analyses. DRB and DMB performed the bioinformatics analyses, sorted data from those analyses and wrote the manuscript. All authors read and approved the final manuscript.

## Supplementary Material

Additional file 1 **Results of a BLASTP search of all annotated *Frankia *transposases**. Excel workbook containing formatted data from a BLASTP search of the non-redundant database using all annotated transposase amino acid sequences from the three *Frankia *strains as a query. Transposase ORFs that had a G+C% content less than 65% have a listing of their G+C percentage in the "G+C%" column. Inverted Repeats identified by the IRF program  are listed in the "Left IR" and "Right IR" columns. Click here for file

Additional file 2 **Results of a PSI-TBLASTN search of the three *Frankia *genomes using five transposase PSSMs**. Excel workbook containing PSI-TBLASTN search results from all three *Frankia *genomes. Click here for file

Additional file 3 **Positions of Large Chromosome Rearrangements in EAN and CcI3**. Excel workbook containing LCR coordinates and transposase contents in strains CcI3 and EAN1. Click here for file
